# Clinical risk factors for osteoporosis are common among elderly people in Nuuk, Greenland

**DOI:** 10.3402/ijch.v72i0.19596

**Published:** 2013-01-11

**Authors:** Anna Jakobsen, Peter Laurberg, Peter Vestergaard, Stig Andersen

**Affiliations:** 1 Arctic Health Research Centre, Aalborg University Hospital, Aalborg, Denmark; 2 Department of Internal Medicine, Queen Ingrids Hospital, Nuuk, Greenland; 3 Department of Endocrinology, Aalborg University Hospital, Aalborg, Denmark; 4 Department of Health Science and Technology, Aalborg University Hospital, Aalborg, Denmark; 5 Department of Geriatric Medicine, Aalborg University Hospital, Aalborg, Denmark

**Keywords:** risk factors, osteoporosis, fragility fractures, Greenland Inuit, old people

## Abstract

**Background:**

Osteoporosis is a debilitating condition characterized by fractures, pain and premature death. Risk factors for osteoporosis predict the risk of fragility fractures.

**Aim:**

To describe the occurrence of risk factors for osteoporosis among populations in Nuuk, the capital of Greenland.

**Methods:**

A random sample of women born in 1934–42, 1945–47, 1956, and men born in 1956 were selected from the national civil registry. A questionnaire was sent out in Greenlandic and Danish on risk factors for osteoporosis: family history, smoking habits, alcohol intake, presence of disease, sun exposure, intake of dairy products, age at menopause (women) and number of falls. Additional questions included the frequency of back pain, previous fractures, intake of vitamin D and calcium supplements, use of anti-osteoporotic drugs, steroids and other drugs.

**Results:**

The questionnaire was sent to 317 subjects confirmed to be living at an address in Nuuk and 181 (57.1%) responded. More young women than older women were smokers (60.6% vs. 35.0%; p=0.022) while limited sun exposure was reported by more of the old women (37.2% vs. 5.6%; p=0.003). Family history of osteoporosis was reported by 15.0%, without difference between groups. Alcohol and milk intake did not differ between groups. Premature menopause was reported by 17.9% of the women. Falls within the last year were reported by 42.4% with fewer falls in the oldest age group (21.9% vs. 50.0%; p=0.005). Frequency of fragility fractures increased with age (5.7% vs. 24.3% vs. 30.4%; p=0.02) and the risk of a fragility fracture increased with age (p=0.004; OR, 95% CI: 4.5, 1.6–12.2, reference: below 70 years), when adjusted for smoking, gender and falls. The use of anti-osteoporotic drugs was low (3.4%) while 28.8% took calcium and vitamin D supplements.

**Conclusions:**

Age is a dominating risk factor for fragility fractures in Greenland. The use of anti-osteoporotic drugs is low in Greenland, even if osteoporotic fractures are common in old age.

Osteoporosis is a condition characterised by low bone density and micro-architectural deterioration of bone tissue, which raises the risk of fracture (1). Fractures are associated with pain, decreased quality of life, disability and premature death and impose considerable costs on society (2–5).

Osteoporosis may be diagnosed when a fragility fracture occurs. These are typically seen at the hip, forearm or lower thoracic and lumbar spine, though fracture risk at other sites is also increased (6–8). It is possible to predict the risk of osteoporotic fractures through the use of risk factor analysis and bone mineral density (BMD) testing. Low BMD is associated with increased fracture risk (9, 10) that may be reduced by treatment (7), and a number of other factors may be used to evaluate fracture risk (7).

The risk of an osteoporotic fracture differs between regions and may thus be influenced by ethnic origin (11–13). Inuit are a distinct ethnic group (14) and their risk of fragility fractures remains to be elucidated. A study of BMD measured by dual energy x-ray absorptiometry (DXA) reported no differences in BMD between Inuit and non-Inuit in Greenland when adjusted for differences in body size (15). However, a key clinical point is to identify individuals at risk, and further evaluation is recommended for those who have a risk factor for osteoporosis (16).

A number of factors are associated with an increased risk of osteoporosis. Surveys of cardiovascular risk factors among Inuit in Greenland have documented frequent smoking and increasing sedentary lifestyle (17, 18). These are known risk factors in other populations and are likely to also increase the risk of osteoporosis among Greenlandic Inuit but a survey of risk factors for this specific population is yet to be conducted.

This led us to conduct a survey on risk factors for osteoporotic fractures among populations in Nuuk, Greenland. In addition, we assessed the intake of calcium and vitamin D supplements, and of anti-osteoporotic drugs.

## Methods

### Participants

Participants were identified through the National Civil Registration system in which all persons living in Greenland, Denmark and the Faeroe Islands are recorded. We aimed to include women aged 75, 65 and 55 years at the time of investigation and selected all women living in Nuuk who were born in 1934–42 and compared them with 2 groups of younger post- and perimenopausal women born in 1945–47 and in 1956. Also, a group of men born in 1956 was included (Table I). We included all 317 subjects in the selected age groups, confirmed to be living on the address in the capital city, Nuuk. The city has approximately 16,000 inhabitants and the number of elderly people is limited due to an average life span in Greenland of 66.6 years for men and 71.6 years for women (19). The oldest age group counted 163 subjects and only 7 (4.3%) of these were not born in Greenland. Some delay was encountered in the updating of the registry and 169 persons had moved away, died or were unavailable at the address.

**Table I:** Number of participants and questionnaire response rate in the osteoporosis risk factor survey in Greenland

Groups selected			Registered^a^ n	Selected^b^ n	Responders	
	
Year born	Age	Gender			n	%
1934–42	69–77 years	Women	163	102	60	58.8
1945–47	63–65 years	Women	117	75	40	53.3
1956	54–55 years	Women	105	68	36	52.9
1956	54–55 years	Men	101	72	45	62.5
All			486	317	181	57.1

a Number of participants recorded in the national civil registration system.

b Number of subjects available at the address.

A cover letter and a questionnaire in both Greenlandic and Danish were sent out in early April 2011 and a stamped addressed envelope was enclosed.

### Questionnaire

The questionnaire included questions related to osteoporosis in other groups. The questions examined family history of osteoporosis with a fracture of forearm, spine or hip, smoking habits (present, past or never), alcohol intake (below or above 14 units/week), sun exposure (limited, average or above average), intake of milk or cheese (daily, weekly or rarely), age at menopause, use of steroids and other drugs or the presence of diseases that may influence bone strength, number of falls within the last week, month and year, frequency of back pain (never, rarely, weekly, and daily), if an x-ray that documented a fracture had been done (spine, hip, forearm and other) with a description of what caused the fracture, a history of hip surgery, daily intake of vitamin D and calcium supplements, and use of anti-osteoporotic drugs. Assessment of osteoporosis by the measurement of BMD was not possible as there is no such scanner in Greenland. Information on age and sex was obtained from the national civil registration system. Beverages fortified with calcium and vitamin D were not available in Greenland.

## Statistical analysis

Frequencies were compared using chi-squared test. Kendall's tau correlation was used to test for associations between groups. Dependent variables entered into logistic regression was an osteoporotic fracture while explanatory variables were age (oldest age group, yes/no), gender, presently a smoker and falls within the last year (yes/no). Also, age, gender and number of risk factors (reference: 2 or less) were entered as explanatory variables. Data were entered into the database using EpiData Software (The EpiData Association, Odense, Denmark, www.epidata.dk). Calculations were performed using the Statistical Package for the Social Sciences (version 13.0; SPSS Inc., Chicago) software. A p-value of less than 0.05 was considered significant.

## Results

The questionnaire was sent to 317 inhabitants in Nuuk in the specific age and sex groups confirmed as living at the address (Table I). The overall response rate was 57.1%.

Table II shows the risk factors for osteoporosis in the 4 groups. One in six reported a family history of osteoporosis and there was a trend towards more frequent family history of osteoporosis with advancing age (p=0.060). The number of present smokers decreased with age (trend, p=0.002) from half of the younger to 1 in 3 of the oldest age group (Table II) and fewer of the younger women were never smokers. One in ten of the oldest and 1 in 5 of the youngest had a high alcohol intake. None of the oldest women took glucocorticoids but 1 in 7 reported diseases that may increase the risk of osteoporosis. Limited sun exposure was reported by 1 in 7 of the younger respondents and by 1 in 3 of the oldest women, and sun exposure declined with age (trend, p=0.009). One in five women reported premature menopause. Family history of osteoporosis, smoking habits, alcohol intake, frequency of disease, use of steroids or other drugs that can affect the bones, and intake of dairy products did not differ with gender.

**Table II:** The occurrence of risk factors for osteoporosis in the study population in Nuuk

	Men		Women							
	
	Aged 54–55 years		Aged 54–55 years		Aged 63–65 years		Aged 69–77 years			
			
	n	%	n	%	n	%	n	%	P^a^	P^b^
Family history										
Yes	4	8.9	4	11.4	6	16.2	11	22.0	ns	ns
No	41	91.1	31	88.6	31	83.8	39	78.0		
Smoker										
Present	19	42.2	20	60.6	16	41.0	21	35.0	ns	0.022
Past	17	37.8	12	36.4	16	41.0	21	35.0		
Never	9	20.0	1	3.0	7	18.0	18	30.0		
Alcohol units/week										
<15	37	82.2	28	77.8	30	76.9	52	89.7	ns	ns
15+	8	17.8	8	22.0	9	23.1	6	10.3		
Other diseases										
Yes	5	11.1	4	11.1	3	7.5	9	15.0	ns	ns
No	40	88.9	32	88.9	37	92.5	51	85.0		
Steroid daily										
Yes	1	2.2	1	2.8	0	0.0	0	0.0	na	na
No	44	97.8	35	97.2	38	100	52	100		
Other bone affecting drugs										
Yes	1	2.2	0	0.0	0	0.0	1	1.8	na	na
No	44	97.8	35	100	38	100	54	98.2		
Sun exposure										
Often	3	6.7	3	8.3	2	5.3	6	11.8	ns	0.003
Some	34	75.5	31	86.1	30	78.9	26	51.0		
Limited	8	17.8	2	5.6	6	15.8	19	37.2		
Dairy products										
Daily	29	64.4	26	72.2	24	63.2	41	73.2	ns	ns
Weekly	7	15.6	6	16.7	5	13.2	9	16.1		
Rarely	9	20.0	4	11.1	9	23.6	6	10.7		
Age at menopause										
<45	na		6	18.8	7	25.0	4	11.4	na	ns
45+	na		26	81.2	21	75.0	31	88.6		

a Chi-squared test for differences between genders.

b Chi-squared test for differences with age between women.

Missing: Family history, 14; smoker, 4; alcohol intake, 3; steroid use, 10; use of drug affecting bone, 8; sun exposure, 11; intake of dairy products, 6; age at menopause, 41.

Fewer women in the oldest age group compared to younger women reported falls (p=0.005). Among women aged 63–65 years, 61.3% reported 1 or more falls within the last year, compared to 46.4% among the youngest and 21.9% among the oldest women (Table III).

**Table III:** The frequency of events associated with osteoporosis and the use of antiosteoporotic drug and calcium and vitamin D supplements

	Men		Women							
	
	54–55 years^a^		54–55 years^a^		63–65 years^a^		69–77 years^a^			
			
	n	%	n	%	n	%	n	%	P^b^
Back pain									
Never	3	7.5	4	12.5	2	6.3	10	23.9	ns
Rarely	28	70.0	22	68.7	17	53.1	19	45.2	
Weekly	6	15.0	3	9.4	3	9.4	5	11.9	
Daily	3	7.5	3	9.4	10	31.2	8	19.0	
Falls (n)									
Last year	11	40.7	13	46.4	19	61.3	7	21.9	0.005
Last month	6	24.0	5	19.2	10	34.5	0	0.0	0.011
Last week	1	3.2	2	6.7	3	10.7	0	0.0	na
Fracture									
None	26	60.4	26	74.3	15	40.5	32	57.1	0.018
Osteoporotic	3	7.0	2	5.7	9	24.3	17	30.4	0.020
Other	14	32.6	7	20.0	13	35.2	7	12.5	
Hip surgery									
Yes	0	0.0	1	2.8	2	5.3	4	7.1	na
No	45	100.0	35	97.2	36	94.7	52	92.9	
Osteoporotic drug									
Yes	0	0.0	0	0.0	2	5.3	4	7.1	na
No	45	100.0	36	100.0	36	94.7	52	92.9	
Vitamin D									
Yes	15	34.1	15	42.9	18	47.4	22	37.9	ns
No	29	65.9	20	57.1	20	52.6	36	62.1	
Calcium									
Yes	12	27.3	11	30.6	13	35.1	21	36.8	ns
No	32	72.7	25	69.4	24	64.9	36	63.2	

a Decades born: 1930s, 1940s, and 1950s.

b Difference between women.

Missing: Back pain, 35; falls, 52; fracture, 10; hip surgery, 6; drug for osteoporosis, 6; intake of vitamin D, 9; and calcium, 7.

**Fig. 1:**
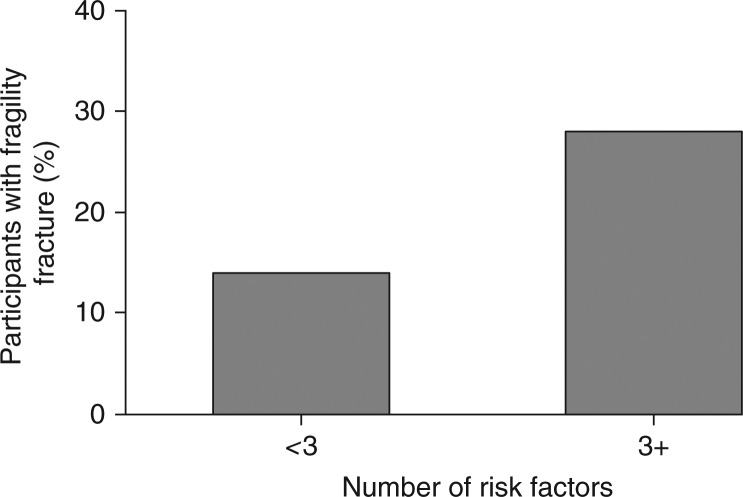
Percentage of participants with a previous fragility fracture depending on number of risk factors for osteoporosis. The risk of a fragility fracture was higher with 3 or more risk factors (p=0.031).

Overall, having 3 or more risk factors for osteoporosis doubled the risk of a previous fragility fracture (Figure 1, p=0.031). This was also found after adjusting for age and gender (p=0.045; OR, 95% CI: 2.3, 1.0–5.3 (1 or no risk factor reference)).

Women aged 63–65 years reported back pain more frequently than the other groups, as 40.6% had listed this “daily” or “weekly”. In comparison 31.0% of women aged 69–77 years, 18.8% of women aged 54–55 and 22.5% of men reported back pain. Back pain was associated with the number of risk factors (p<0.001) and with the occurrence of fragility fractures (p=0.013).

**Fig. 2:**
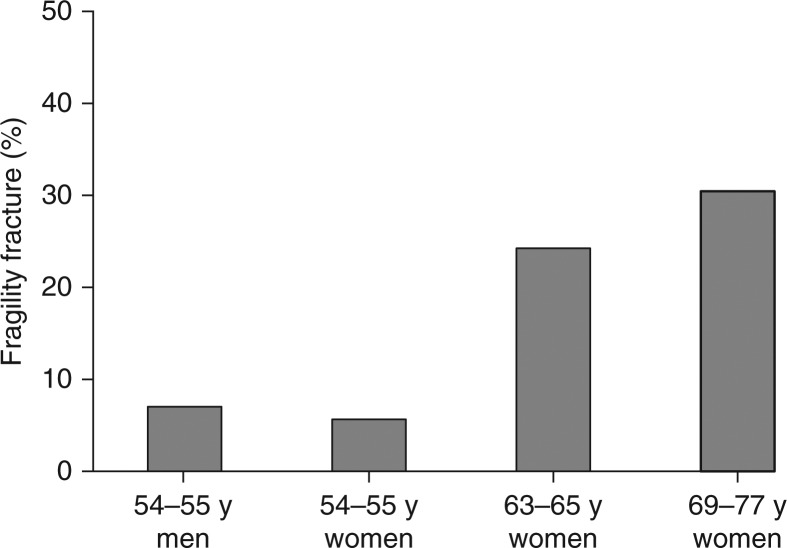
Percentage of participants who had fragility fracture by participant groups. The occurrence increased with age (p=0.009).

Fragility fractures were reported more frequently with increasing age (p=0.009) as depicted in Fig. 2. Hip fractures were reported by 3 women aged 70 years or above. Also, 1 man reported a hip fracture before the age of 30 years. Age remained a main risk factor for an osteoporotic fracture in the adjusted analysis (p=0.005; OR, 95% CI: 3.2, 1.4–7.2 (age <69 years reference)).

Four women aged 69–77 years and 2 aged 63–65 years took an anti-osteoporotic drug (Table III). None of the women aged 54–55 or the men used such drugs. Four of the participants who took an antiresorptive drug reported no fragility fractures while only 1 with a forearm fracture and 1 with a vertebral fracture reported the use of anti-osteoporotic drugs. Two (6.5%) of those who had previously experienced an osteoporotic fracture were currently taking medication for osteoporosis.

Finally, “any fracture” was associated with high alcohol intake (p=0.043) and back pain (p=0.037) as well as a “number of risk factors” (p=0.003).

## Discussion

Risk factors for osteoporosis are common among populations in the capital city Nuuk in Greenland and increase the risk of having had an osteoporotic fracture as 1 in 3 women in the oldest age group reported to have suffered from at least 1 fragility fracture verified by x-ray and subjects with multiple risk factors for osteoporosis had an increased risk of fractures. Only 2 of the participants with a fragility fracture, and 6 in the entire survey, took an antiosteoporotic drug and focus on osteoporosis is encouraged.

Fracture rates have been reported to differ with ethnic origin (12, 13). The number of hip fractures was lower in African-Americans compared with American Whites (20–22). Bow et al. (23) found that Asians aged 65 years or above suffered only half the number of hip fractures compared to Caucasians, but at least as many vertebral fractures, which is in keeping with findings by others (24). Thus, studies comparing Caucasians, African-Americans and Asians suggest that ethnicity influences the risk of osteoporotic fractures.

A few studies of osteoporotic fractures have been made among Inuit. A study from Alaska reported twice the number of hip fractures in Inuit compared to subjects from the lower states in the United States (25). However, this study did not take into account environmental differences such as the number of days with icy pavements, the number of hours with sunlight and the presence of a polar night, which may influence the risk of falls. These are considered risk factors for osteoporosis, as up to 98% of hip fractures are related to falls (26). Leslie et al. (27) found aboriginals in Canada to suffer twice as many osteoporotic fractures compared to non-aboriginals after adjusting for gender, age and area of residence. They used administrative health data and explained their finding of a higher fracture risk by co-morbidity and substance abuse rather than by ethnicity. The responders in our survey confirm more frequent fragility fractures among women aged 63–65 years and 69–77 years compared to younger women in the capital city of Greenland. This finding is similar to those in other groups and suggests some similarity between populations in Greenland and elsewhere.

Osteoporotic fractures are more frequent in subjects with low BMD (9, 10). Nelson et al. (28) found that differences in BMD between ethnic groups in 2 countries were smaller than the differences between different ethnic groups within the same country. While this supports an ethnic difference in BMD, a comparative study of Inuit and non-Inuit in North Greenland found similar BMD in the 2 ethnic groups when adjusted for body build (15). Hence, no ethnic difference in BMD seems to be present between Inuit and Caucasians.

Factors important for the risk of osteoporosis among other ethnic groups (7, 16) may also play a role among populations in Greenland. This is suggested by the findings by Cote and colleagues (29, 30). They measured bone stiffness by bone ultrasound and found an association with age, smoking, menopause, use of hormone replacement therapy, physical activity, and weight and height in addition to an influence of polychlorinated biphenyls. Our finding that fragility fractures are twice as common in individuals with more than 2 risk factors lends further support to this notion.

Smoking is a well-established risk factor for osteoporosis in other populations (30, 31) and the impact of smoking may increase with latitude (32). Accordingly, Filner and colleagues (33) found that current and former smoking increased the risk of low BMD in Alaska natives. In our survey, 61% of the younger respondents were smokers, which is in keeping with previous findings (17). Fewer in the old age group were current smokers but the old responders remained at a higher risk of osteoporosis compared with never smokers. Hence, smoking may be an important factor in influencing fragility fractures in Greenland but the size of the population studied did not allow for analysis of individual risk factors.

Limited alcohol consumption may have a positive influence on BMD while heavy drinking increases the risk of osteoporotic fractures (34, 35). In our survey, alcohol consumption was associated only with the risk of any fracture and not with fragility fractures. This may relate either to the drinking pattern in Greenland that is characterised by binge drinking or to the lack of statistical power due to the limited number of heavy drinkers in our survey.

Osteoporosis is a common disease at high latitude countries such as the Scandinavian countries (11, 36). This may relate to low vitamin D levels due to inadequate sun exposure. The solar zenith angle is even higher in Greenland than in Scandinavia. Still, we included the question “limited sun exposure” in our questionnaire and we consider it to be a risk factor because the high intensity of the light in Greenland during spring and indication of dermal vitamin D production even in North Greenland (37) in addition to vitamin D provided by the traditional Inuit diet (38). Furthermore, stratospheric ozone depletion increases the UVB radiation in the circumpolar area in the range required for dermal vitamin D production (39). Thus, sun exposure is likely an issue in populations in Greenland too.

Relatively few participants reported a family history of osteoporosis compared to other populations (7). The low number may be due to a lack of awareness of osteoporosis in Greenland as the disease manifests with fractures at old age. A short life span was common until recent years. The mean life span has increased by 5.9 and 3.2 years among men and women respectively over the past 18 years and the fraction of people aged 65 years and older is expected to double in the next 30 years (40). This will increase the occurrence of fragility fractures and hence the awareness of osteoporosis.

Age is a major risk factor for osteoporosis in other populations (30, 31, 41). A similar association was seen in our study with a rise in fragility fractures with age leading to an increase in fragility fractures that reached 1 in 3 of the oldest women. Hence, focus on osteoporosis in Greenland is important considering the predicted increase in life expectancy.

The number of falls was markedly lower in the oldest compared to the younger age groups. This may be explained by the fact that the oldest people are retired and don't have to go out on icy pavements and roads combined with an inability to get outside when conditions are inhospitable. This is in keeping with the notably lower sun exposure reported by the oldest age group. Despite the fewer falls in the old, there was a steep rise in the occurrence of fragility fractures with age, which emphasises the importance of focus on both the treatment and prevention of osteoporosis in Greenland.

Treatment of osteoporosis was reported to be very scarce. Even subjects with obvious fragility fractures did not take medication for osteoporosis. This seems odd as all medication in Greenland is free of charge and anti-osteoporotic drugs are efficient and readily available. Adherence to treatment with anti-osteoporotic drugs may be low due to side effects. Still, the low frequency of treatment is common in many countries and should be addressed in Greenland.

Our survey had limitations. First, the study population was of limited size. However, we included all inhabitants in the capital Nuuk in the defined age groups and had to extend the age range in the oldest group to reach an acceptable number of participants. Despite the limited size, we were able to reach valid conclusions regarding the importance of factors that are associated with an increased risk of osteoporosis in other ethnic groups: Advanced age, smoking, limited sun exposure, falls, the occurrence of previous fractures and the “number of risk factors”. Second, a delay in the recording of address changes combined with a frequent change of address may have influenced risk profiles of the participant groups as those who have a frequent change of address or have no permanent address differ in income and social group compared to those with a more stable life style, and the former group carries a higher risk of osteoporosis. They are less likely to have a registered address and they may not have responded. This tends to underestimate the risk of osteoporosis found in our survey. Third, the questionnaire survey carries the risk of reporting bias and findings should be confirmed by surveys using other methods.

Confirming the occurrence of risk factors for osteoporosis in populations in Greenland helps to prevent future cases of osteoporosis and fractures. In doing so, we hope to reduce the pain, decreased quality of life, disability and premature death, and the considerable costs that this disorder places on the Greenlandic society (2–5).
